# Simultaneous identification of animal-derived components in meats using high-throughput sequencing in combination with a custom-built mitochondrial genome database

**DOI:** 10.1038/s41598-020-65724-4

**Published:** 2020-06-02

**Authors:** Yinan Zhang, Qinfeng Qu, Mingzhen Rao, Nana Zhang, Yu Zhao, Fei Tao

**Affiliations:** 1 Shanghai Institute of Quality Inspection and Technical Research, Shanghai, 200233 People’s Republic of China; 2 State Key Laboratory of Microbial Metabolism, and School of Life Sciences & Biotechnology, Shanghai Jiao Tong University, Shanghai, 200240 People’s Republic of China; 3 College of Life Science, Shanghai Normal University, Shanghai, 200234 People’s Republic of China

**Keywords:** Assay systems, Next-generation sequencing

## Abstract

Currently, the inspection and supervision of animal ingredients relies primarily upon specific amplification-dependent methods, whose efficiency and accuracy are being seriously challenged by the increasing diversity and complexity of meat products. High-throughput sequencing (HTS) technology was employed to develop an alternative method to detect animal-derived ingredients in meat products. A custom-built database containing 2,354 complete mitochondrial genomic sequences from animals, an identification analysis pipeline based on short-sequence alignment, and a web-based server were built to facilitate this detection. The entire process, including DNA extraction, gene amplification, and sequencing, was established and optimized for both marker gene (part of the *CYTB* gene)-based detection and total DNA-based detection. Using simulated samples containing various levels of pig, cattle, sheep, chicken, rabbit, and mice ingredients, the detection capability and accuracy of this method were investigated. The results of this study indicated that the method is capable of detecting animal components in meats that are present at levels as low as 1%. Our method was then tested using 28 batches of real meat products such as raw meat slices, raw meat mince, cooked dried meat, cooked meat sausage, and other supermarket samples, with a traditional qPCR method as the control. The results demonstrated an accuracy of 97.65% for the qualitative detection method, which indicate that the developed method is reliable for the detection of animal components. The method is also effective for the identification of unknown food samples containing mixed animal components, which suggests a good future in application.

## Introduction

The number of deep-processed meat products is steadily increasing, and consumers can no longer accurately judge the real ingredients of meat products by relying solely on the particular texture and flavor of meat products. Adulteration and fraudulent labeling of commercial meat products occur frequently, and this can result in a number of health risks and even moral and religious problems^1^.

Traditional analytical methods and assays, such as enzyme-linked immunosorbent assay (ELISA), liquid chromatography (LC), isoelectric focusing electrophoresis (IFE), and PCR, are often insufficient to identify the unknown and multiple species of origin in meat products^1,2^. Therefore, more effective alternative methods are in high demand, and potential methods have been investigated intensively. For example, specific multi-PCR (mPCR) and/or random PCR followed by the subsequent amplicon analysis that include SNP (Single Nucleotide Polymorphism), RFLP (Restriction Fragment Length Polymorphism), and RAPD (Random amplified polymorphic DNA) have been reported extensively. The resolution, universality, and scalability of these reported methods, however, are still unsatisfactory, particularly for complex samples^3^. With the development of high-throughput sequencing, the DNA meta-barcoding approach has emerged as a promising method for the identification of unknown and multiple species^3^. This method has been investigated extensively for the identification of organisms in the environment^4^, characterization of traditional Chinese medicines^5^, diet assessment^6^, and fisheries applications^7^.

Genes harbored within animal mitochondria are considered to be high-quality targets for food identification due to their high copy numbers and abundant phylogenetic information. The *CYTB* gene is a typical mitochondria gene that contains phylogenetic information extending from the intraspecific level to the intergeneric level^8^, and it has been used as a common target gene for meat component identification in a number of studies. In recent decades, several applied studies using the mPCR or PCR-RFLP methods have been performed with the *CYTB* gene as a target to identify meat components of up to approximately 20 species^9–13^. The D-loop region, *COI* gene, and rRNA genes are also good targets for species identification, and they have been reported to be effective for food identification^14–16^. These studies presented the advantages and potential of the identification methods that are based on mitochondrial genetic information.

As well known, universal primers are important because they determine the amplicons of which the sequences are critical for molecular taxonomy. In 1989, Kocher designed a pair of universal primers, CB1-5 and CB3A, to amplify the *CYTB* gene for investigating the evolutionary dynamics of animal mitochondrial genomes. These primers can amplify 221 sequences from 106 species of animals, including mammals (rodents, carnivores, hoofed, primates, sloths and marsupials), poultry (songbirds, wild birds and waterfowls), amphibians (salamanders and frogs), reptiles (crocodiles), and fish (sharks, lilies and salmon)^17^. In 2004, Bravi *et al*. conducted RFLP-PCR using these primers to identify cattle, horses, donkeys, pigs, sheep, dogs, cats, rabbits, chickens, and humans^18^. In 2009, Murugaiah *et al*. used similar methods to identify pigs, cows, buffalo, chickens, goats, quails, and rabbits^19^. In 2003, Verma and Singh conducted an empirical analysis for wildlife identification using amplicon sequencing, and they successfully identified 221 animal species without knowing the sample composition^20^. These reports strongly suggest that the primers CB1-5 and CB3A are promising candidates for meat identification.

With the development of sequencing technology, particularly the emergence of next-generation sequencing (NGS), and bioinformatic methods for massive data analysis, sequencing-based methods for DNA barcode region identification is becoming increasingly promising. Tillmar *et al*. successfully identified mixed DNA from different mammals using a 454 GS Junior sequencer with mtDNA 16S rRNA gene as the target^21^. In 2014, a method named All-Food-Seq (AFS) was reported as useful for unknown component analysis, a technique that relies on genome-wide DNA in-depth sequencing and analysis and is more accurate, qualitative, and quantitative^22^. Both of these reported sequencing-based methods, however, are not popular for routine detection practice due to their high demand for analysis and sequencing data volume.

In this study, a custom-built database containing 2,354 complete mitochondrial genome sequences was developed for identification of animal components in food. The high throughput sequencing procedures and data analysis pipeline based on an advanced short sequence matching algorithm were built and optimized. A web server was also built for facilitating the analysis. Both the target-gene directed method and the All-Food-Seq method were developed and systematically tested. The developed method was also compared to the traditional method to determine its accuracy. The developed method is promising for the identification of animal-derived ingredients in food, and this method is ready for commercial application in meat product inspection.

## Results

### PCR amplification and the phylogenetic analysis of the amplicon

**Figure 1:**
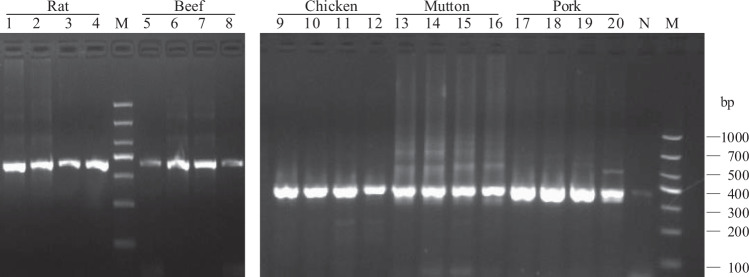
PCR amplification using universal primers. Agarose gel electrophoresis were performed to evaluate the amplification of *CYTB* amplicon using universal primers. The labels Rat, Beef, Chicken, Mutton, Pork, represents the different origins of amplification templates. The lanes: 1–4, rat; 5–8, beef; 9–12, chicken; 13–17, Mutton; 18–21, pork; 22, Negative control; M, Marker DL 1000. There are 4 lanes for each meat sample, which represent different annealing temperatures of amplifications. The annealing temperatures are 50 °C, 55 °C, 60 °C, and 65 °C from left to right. The electrophorograms of the rat amplicons, the beef amplicons and the three other meat amplicons cropped from different gels, which were separated with white space. The raw full-size gels were also displayed in Fig. S1(c).

PCR amplification at annealing temperatures of 50 °C, 55 °C, 60 °C, and 65 °C was performed using universal primer pairs for CB1-5 and CB3A, the COIF and COIR, and the CE CVZV 1 and CE CVZV 2, respectively (Table 1). The amplification products were analyzed by agarose gel electrophoresis. The primer pairs of CB1-5 and CB3A were more universal than the other two primer pairs, as a single band of approximately 385 bp was amplified from chicken, sheep, pig, rat, cattle, and rabbit genomes using the *CYTB* gene derived primer pairs (Fig. 1). The primer pairs of *COI*^23^ didn’t work for chickens, and the pairs of D-loop didn’t work for pork (Fig. S1). Given this, the primer pairs of CB1-5 and CB3A with an annealing temperature of 55 °C were selected for subsequent amplification.

**Table 1:** Primers.

Target gene	Primer Code	Sequence (5′–3′)	Primer sources
*COI*-5P	COIF	AATTGGGGGGTTTGGAAATTG	^23^
	COIR	GCTCGTGTATCAACGTCTATTCC	
*D-loop*	CE CVZV 1	GATCACGAGCTTGATCACCA	www.apvv.sk, PROJECT APVV-0368-10
	CE CVZV 2	AGGAGTGGGCGATTTTAGGT	
*CYTB*	CB1-5	CCATCCAACATCTCAGCATGATGAAA	^8^
	CB3A	CCCTCAGAATGATATTTGTCCTCA

**Figure 2:**
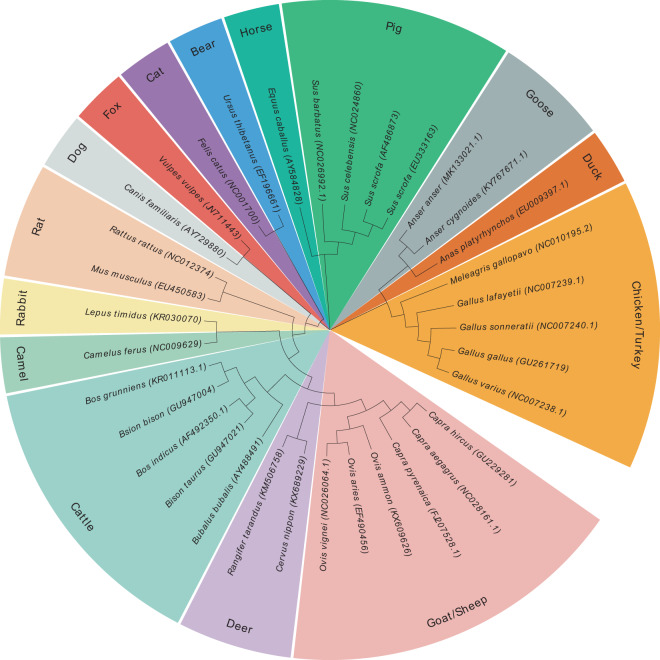
Phylogenetic tree analysis of *CYTB* amplicons. The evolutionary tree was constructed according to the ***CYTB*** amplicon sequences. The tree was constructed using MEGA-X by neighbor-joining method with Maximum Composite Likelihood and decorated with EvolView. Each leaf node represents a simulated amplicon derived from a whole mitochondrial genome obtained from the NCBI using the universal primer pairs CB1-5 and CB3A. The representative species names including accession numbers composed the sets of leaf labels. The length of the clades presents the evolutionary distance. Species belong to a meat type are represented using the same background color.

To evaluate the identification potential of the amplicon, 34 species of domestic animals, special livestock and poultry, such as cattle, buffalo, goats, sheep, sika deer, reindeer, rabbits, camels, pigs, horses, cats, bears, dogs, foxes, chickens, rats, and mice, were selected to construct the phylogenetic tree (Fig. 2). The 34 corresponding sequences used for tree building were sliced from the mitochondrial whole genome sequences in NCBI database with the above universal primers as terminals. From the constructed phylogenetic tree and the corresponding taxonomic nomenclature, one can observe that the sequence contains both intraspecific and interspecific evolutionary information, which is critical for classification. The branches in the phylogenetic tree are consistent with the generic taxonomy of animal species and the corresponding kinds of meats. This result suggests that the selected universal primer sequences are excellent in defining PCR amplicons for identifying the meats derived from the domestic animals, special livestock, poultry, games, and even non-food-producing animals.

### High throughput sequencing

Mixed DNA sample H1 was sequenced on the high throughput Illumina HiSeq while the samples SP-1, SP-2, and SP-3 were sequenced using MiSeq platform. There are 1,764,067 reads being produced for H1, with a read length of 151 bp. There are 341,370, 368,732, and 356,172 reads being obtained for the sample SP-1, SP-2, and SP-3, respectively, with a read length of 301 bp. Then the sequencing qualities of both samples were evaluated using the software FastQC. The quality evaluation items such as Per base sequence content, Per sequence GC content, Sequence duplication levels, and Overrepresented sequence were specially designed for whole genome and meta genome sequencing. Therefore, they were ignored while all other items can pass the quality checking, which indicates that the sequencing outputs were applicable for the subsequent bioinformatic analysis. The average depth of coverage of H1 is 115,313 ×. The average depths of coverage for SP-1, SP-2, and SP-3 are 44,482 ×, 48,047 ×, and 46,410 ×, respectively.

### Database construction and pipeline development

After filtering, we obtained a custom-built database containing 2,354 complete mitochondrial genomes. The genomes distribute on 13 categories including almost all the well-known food producing animals, game animals and meat adulteration related non-food producing animals (Fig. S3). There are 465 mitochondrial genomic sequences for beef while only 14 sequences for camel. This variation is highly consistent with the different research levels of different animal categories.

**Figure 3:**
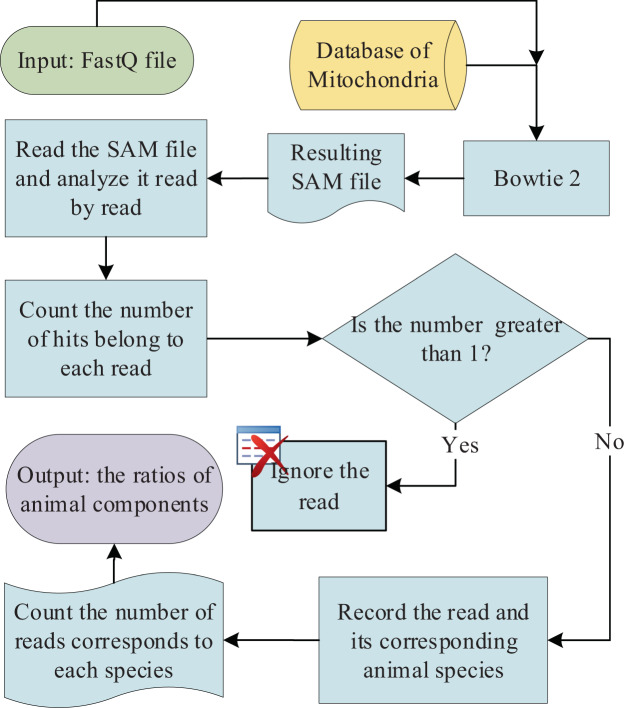
Flow-chart of bioinformatic analysis pipeline. The analysis flow begins from inputting the sequencing data. Then input was compared and mapped to the mitochondrial genomes deposited in our specific database (the yellow block) using the software Bowtie 2.0 with the default parameters. The output SAM file is then parsed with a line-by-line manner. There are usually multiple hits for a single read. The software then checks if the multiple hits of a read belong to the same type of meat. The reads which can map to different meats are discarded, because it can lead to inaccuracy of identification. Only the reads corresponding to one single meat are kept for the following statistical analysis. After finishing the SAM file parsing, the program counts the numbers of reads, which corresponding to different meats, and then outputs the final result in a CSV format file. The rectangle possessing a round head represents the start (green) and end (purple), the yellow block represents the data, the rectangle represents the process, and the diamond represents a decision step. The analysis was programed using Perl language which is run on Ubuntu 16.04 LTS.

To carry out the identification analysis, a standard pipeline was established and briefly described as follows (Fig. 3). Firstly, the obtained sequencing reads were mapped to our custom-built mitochondrial genomic database using the short sequence matching algorithm encoded by Bowtie (2.2.2.9)^24^ to obtain the output SAM file. Secondly, the SAM file was parsed to find all the unique reads which can exclusively aligned to genomes belong to a single meat category (Fig. 3). Simultaneously, the numbers of reads which can aligned to a meat category was also counted up. Then the percentage of each meat category was calculated based on the read numbers and output in a file. For doing this automatically, a Perl script was written (Script S2). Typically, the analysis is expected to take only 5–15 min of computer time, depending on number of reads and sequencing platform. A web server was also developed for making the identification analysis being available for broader users (http://mcii.sjtu.edu.cn). With the web server, one can upload fastq files and complete the identification analysis online. The analysis result will be sent to user through email within 5–15 min upon submission.

### Identification analysis of the artificial mixed DNA sample

To test our analysis method, resulting sequencing files of H1 was analyzed. The analysis result showed that there were 7 meat categories being detected. The varieties of pig, sheep, rat, rabbit, and cattle possessing high matching numbers were in accordance with the varieties preseted for sample H1. So this method is applicable for qualitative DNA analysis. According to the matching number in the file, the percentage content of each kind of meat was calculated and compared to the true value of the artificial samples (Table 2). The results revealed that the matching number of pig ingredients and chicken ingredients with low DNA percentage (2.54% and 1.08%, respectively) were 35,332 and 7,865, respectively, and the percentages of the matching numbers were 3.04% and 0.68%, respectively. As the mean absolute difference between the measured value and the true value in samples is 0.16%–16.98%, and the relative difference is 7.85%–74.80%, this method is not applicable for quantitative identification. According to the results of primer pairs matching analysis (Fig. S1), the differences in binding efficiency of universal primers for various species result in the observed differences in amplification efficiency, ultimately leading to a large degree of error in quantitative analysis. Specifically, the sequences with low amplification efficiency are diluted in the amplification process, and the sequences with high amplification efficiency increase content accordingly.

**Table 2:** Comparison of component contents and sequencing results in sample H1.

Ingredient	Target value of Sample H1		Sequencing result		Difference Abs. (%)	Difference Ref. (%)
	DNA (ng/µL)	Proportion (%)	Reads mapped	Proportion (%)		
Mutton	149.83	40.38	340607	29.28	11.10	27.49
Beef	84.45	22.70	461551	39.68	16.98	74.80
Rabbit	83.13	22.40	200063	17.20	5.20	23.21
Rat	40.17	10.83	116047	9.98	0.85	7.85
Pork	9.46	2.54	35332	3.04	0.50	19.69
Chicken	4.01	1.08	7865	0.68	0.40	37.04
Venison	0	/	1854	0.16	0.16	/
Horse	0	/	1	0.00	/	/
Camel	0	/	1	0.00	/	/
Bear	0	/	1	0.00	/	/

The analysis also indicated that there was a false positive component for venison. After analyzing the output sequence by blast alignment, the similarity between the false positive sequence and the *CYTB* gene sequence obtained from mutton (*Capra, Ovis, Pseudois*) or venison (*Cervus, Capreolus, Odocoileus, Muntiacus, Mazama, Rusa, Hydropotes*) both reached 99%. As each sequence of this high-throughput sequencing possesses only a 151 bp read length, the difference in sequence information used to distinguish mutton and venison is not sufficient. Given this, 300–400 bp read lengths were selected for subsequent high-throughput sequencing analysis to ensure more sequence information to reflect the differences in the varieties.

### Optimization for sequencing throughput

**Figure 4:**
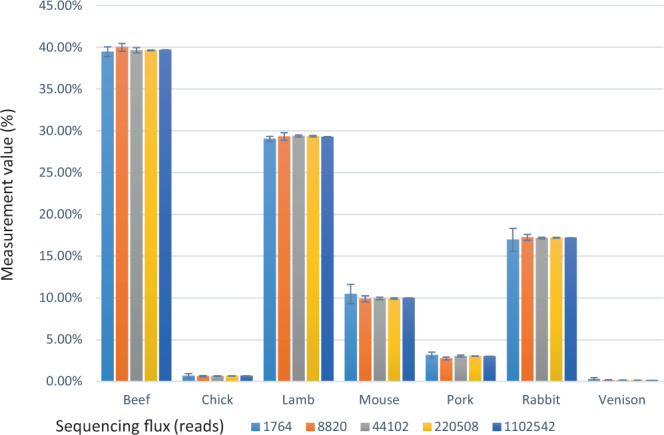
Correlation between sequencing throughput and accuracy. A simulative strategy was used for investigating the effect of sequencing throughput on identification accuracy. A Perl script was used for randomly extracting reads from a fastq file. Different extraction ratios (0.10%, 0.50%, 2.50%, 12.50%, and 62.50%) was used for extracting reads from the 1,764,067 reads of H1. Then the extracted reads were used as data input in the identification process. Each ratio was repeatedly used four times to determine the average values and corresponding standard deviations.

For investigating the effect of sequencing throughput on identification, a series of fastq files were generated by randomly extracting reads from the sequencing result of H1. The analytic results from the simulative sequencing series are shown, and the acceptable sequencing throughput was evaluated according to the standard deviation (Std. Dev.) and relative standard deviation (RSD) (Fig. 4). At sequencing throughput of 1,764 reads, the RSD values of the 1.08% (m/m) chicken DNA, 2.54% (m/m) pork DNA, and 10.35% (m/m) mouse DNA were 43.87%, 10.85%, and 11.07% respectively, and these were not acceptable for biological sample detection. Therefore, at sequencing throughout of 8,820 reads (i.e. the 0.5% extraction ratio of H1 sequencing flux 1,764,067), the RSD value of the method was acceptable for detection. To ensure the reliability of the analysis, the suggested optimal sequencing throughput of this method is approximately 30,000 reads, which greatly reduces the sequencing throughput compared to that of traditional high-throughput sequencing, ultimately reducing the detection cost and making it easier to popularize and apply this method. The following market samples were analyzed using approximately 30,000 reads sequencing throughput.

### Simulated sample detection

To further simulate the real inspection practice, three mixed meat samples SP-1, SP-2, and SP-3 containing different proportions of mutton and pork were prepared to extract total DNA. Both of two kinds of meat were successfully detected. There were no false positive result, such as venison ingredients, been found (Table 3). This result indicated that the method can be used for the qualitative detection of various animal-derived ingredients in mixed meat products.

**Table 3:** Comparison of component contents and sequencing results in samples SP-(1–3).

Sample information		Detection results
ID	Mass percentage (%)	Ingredients/percentage of mapped reads (%)
SQI-SP-1	Sheep 98.95;	Mutton/92.85;
	Pork 1.05	Pork/5.19
SQI-SP-2	Sheep 90.38;	Mutton/69.83;
	Pork 9.62	Pork/28.80
SQI-SP-3	Sheep 48.44;	Sheep/23.07;
	Pork 51.56	Pork/76.22

### Commercial sample detection

The detection of commercial samples was conducted to verify this method in practice conditions. Comparing with the national standard fluorescence quantitative PCR (Table 4), and the true positive rate of the results of high throughput sequencing were 97.56%, the false positive rate were 9.76%, the false negative rate were 2.44%. According to the name of the sample and the ingredient table (Table S2), some of the false positive results of animal ingredients were consistent with those of the animal ingredients listed in the sample ingredient table. Based on our findings, this method is suitable for raw meat slices, raw meat salted, and deeply processed meat (such as smoked and cooked meat, sauced and brine meat, dried meat, etc.). For raw meat samples (De-(1–6) stored at room temperature for 6 days, the matching number is stable, and the results are consistent despite the presence of interference factors such as DNA degradation and microbial increment.

**Table 4:** The comparison of NGS and Fluorescence PCR detection results for commercial meat products.

Sample ID	Meat products	Detection results of	
		NGS	Fluorescence PCR
SQI-P1-1	Beef slices	Beef+	Bovine+
SQI-P1-2	Lamb slices	Mutton+	Ovis+
SQI-P1-3	Mutton rolls	Mutton+	Ovis+
SQI-P1-4	Lamb rolls	Mutton+, Pork+	Ovis+, Porcine-
SQI-P1-5	Mutton rolls	Mutton+, Chicken+	Ovis+, Chicken+
SQI-P1-6	Mutton slices	Mutton+	Ovis+
SQI-P2-1	Ham sausage for noodle soup	Chicken+, Pork+	Chicken+, Porcine+
SQI-P2-2	Chicken ham sausage	Chicken+, Pork+	Chicken+, Porcine+
SQI-P2-3	Ham sausage	Pork+, Chicken+	Chicken+, Porcine+
SQI-P2-4	Spicy and crispy ham sausage	Chicken+, Pork+	Chicken+, Porcine+
SQI-P2-5	Beef-flavored Muslim Sausage	Chicken+, Beef+	Chicken+,
SQI-P2-6	Flavored chicken sausage	Chicken+	Chicken+
SQI-De-1*	Beef rolls	Beef+	Bovine+
SQI-De-2*	Beef rolls	Beef+	Bovine+
SQI-De-3*	Beef rolls	Beef+	Bovine+
SQI-De-4*	Beef rolls	Beef+	Bovine+
SQI-De-5*	Beef rolls	Beef+	Bovine+
SQI-De-6*	Beef rolls	Beef+	Bovine+
SQI-3-1	Beef stick	Beef+	Bovine+
SQI-3-2	Satay flavor beef jerky	Beef+	Bovine+
SQI-3-3	Spicy beef jerky	Beef+, Pork+	Bovine+, Porcine-
SQI-3-4	Pork floss	Pork+, Chicken+	Porcine+, Chicken+
SQI-4-1	Stewed chicken	Chicken+, Pork+	Chicken+, Porcine-
SQI-4-2	Baked Pork Chops	Pork+	Porcine+, Chicken+
SQI-4-3	Lunch leg sausage	Pork+, Chicken+	Porcine+, Chicken+
SQI-4-4	Sliced ham	Pork+	Porcine+
SQI-4-5	soy sauce spiced pork	Pork+, Chicken+	Porcine+, Chicken+
SQI-4-6	American ham	Pork+	Porcine+

Note: *DNA was extracted everyday from the minced comercial beef rolls which were allowed to stand at room temperature for 6 days;+ for positive results, − for negtive results.

## Discussion

With regard to taxonomy, the commonly used DNA barcode *COI* and *CYTB* genes both possess suitable length and a slow evolution rate, allowing them to be used as DNA identification targets. Previous application studies have indicated that although *COI* gene barcode fragments perform well for species identification^25–28^, DNA barcode technology based on it possesses many limitations and shortcomings in the identification of poultry and livestock meat products. First, the DNA barcode sequences of poultry and livestock within the database are not abundant enough and lack supporting data^29^. Second, for some groups of mitochondrial gene fragments, an overlap between intraspecific and interspecific variations still exists. These mitochondrial gene fragments possess limited ability to determine taxonomic levels above that of species^26^. The *CYTB* gene possesses abundant information that can be extended from the intraspecific to the intergeneric level^8^. Therefore, it is a common gene sequence used for meat component identification. Although other genes being useful for species identification were reported, including 12S rDNA, 16S rDNA^30^, tRNA^11^, and D-loop^14^, the *CYTB* gene remains the most advantageous. This method does not require different primer pairs to apply to specific species, and instead, a screened a pair of a DNA barcode universal primers to amplify an aim sequence from the *CYTB* gene, which contains evolutionary information of genus level and above, and can be used for species identification of various food producing poultry and livestock meat.

Through elaborately designed targeted enrichment strategies, novel clonal amplification technology, and chemical sequencing platforms, NGS has facilitated high throughput DNA sequencing at an unprecedented rate and for a reasonable cost. This technology has been used to promote the study of genomics by enhancing several factors, including read length, throughput, read accuracy, read depth, and cost per base^31^. For our study, PCR enrichment products using universal primer sequencing allowed us to obtain 30,000 reads with a length of 300 bp (1 M to 2 M data), and these parameters were necessary for our detection limits of 1% (m/m) and our observed accuracy. Given this, the Illumina MiSeq mentioned and the Ion Torrent sequencing system could be potentially useful for obtaining sequence data for species identification. It is notable that the MinION of Oxford Nanopore Technologies has emerged as new low throughput sequencing technology which can be achieved with portable device and much lower cost. Theoretically, this sequencing technology can well fulfill all the requirements of our method, including the throughput and accuracy. Therefore, it will be highly expectable that the commercial promotion of nanopore sequencer might make our method more promising in the future.

Due to the differences between the quantity of mitochondrial template and the efficiency of PCR amplification, the quantitative accuracy of this method requires improvement. Targeted sequences from single copy genes, the introduction of correction factors for primer amplification efficiency, designing degenerate primers, and strictly controlling the number of amplification cycles and amplification conditions may be useful for solving these quantitative problems.

**Figure 5:**
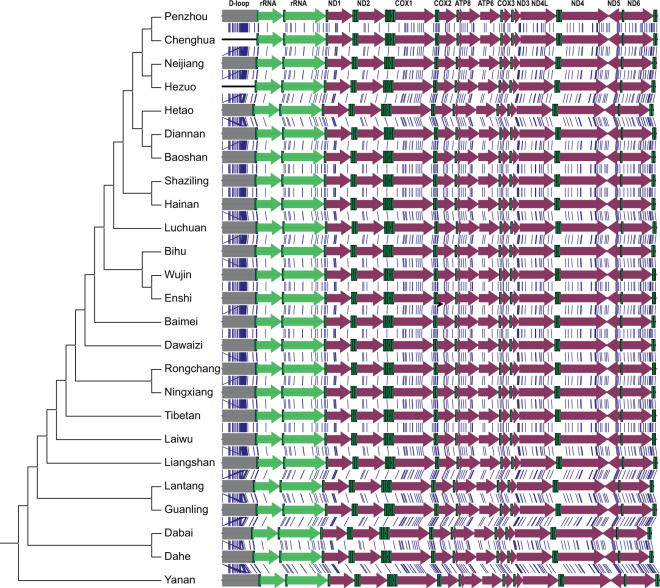
Comparison analysis of mitochondrial genomes of *Sus scrofa*. Twenty-five complete *Sus scrofa* mitochondrial genomes were aligned. Cluster and SNP analysis were performed using MEGA-X. The rRNA genes are presented in light green, D-loop regions are presented in gray, tRNA genes are presented in dark green, and other genes are presented in hot pink. Arrows indicate the gene orientation. The blue lines indicate the positional correspondence of the SNPs. ND1, ND2, ND3, ND4L, ND4, ND5, and ND6 encode the NADH-ubiquinone oxidoreductase chain 1–6 and chain 4L, respectively. COX1, COX2, and COX3 encode cytochrome c oxidase subunit 1–3, respectively. ATP8, ATP synthase F0 subunit 8; ATP6, ATP synthase F0 subunit 6.

It must be noted that complete mitochondrial genome sequences were used to build our database. This will allow our database and the corresponding web-server to perform identification based on other mitochondrial genes or on the whole genome sequence. Theoretically, it will be possible to identify the breeding species of animals using the developed database and web server if using a multiple gene or complete mt-genome approach. In fact, we did a brief test by using a simulate sample of mixing meat (Table S3), which initially suggested the feasibility of the developed method. To further investigate this potential, we selected the complete mt-genome sequences of 25 *Sus scrofa* breeds that were used for pork production and performed sequencing analysis (Fig. 5). Our results indicated that the breeds that are closely related can be clustered together, suggesting that the mt-genome sequences can be used to determine the relationship between different *Sus scrofa* breeds. Figure 5 also clearly indicates that a large number of SNPs exist and are distributed throughout all mitochondrial genes, suggesting a high potential for breed-level identifications based on complete mt-genome sequencing or multiple-gene sequencing. The analysis also identified other regions possessing a high density of SNPs, such as the D-loop region. These regions will be preferential candidates for the development of future identification methods.

In summary, by combining high-throughput sequencing, a specific mitochondrial genome database, and an established bioinformatic pipeline, a comprehensive analytical method was developed to allow for identification of animal-derived components. The method can efficiently and accurately identify animal components using data produced by target-gene amplicon sequencing. The specific database contains 2,354 mitochondrial genomes covering the majority of animals, and this provides our method with promising potential for unknown component analysis. This will provide advantages for the analysis of animal raw materials within food containing unknown complex ingredients, a process that is critical for identifying adulteration of raw materials, detecting allergen components, tracing material sources, and safety risk warning in food safety emergencies. This novel method enabled simultaneous identification of original animal components and possesses the potential for widespread use in food inspection practice.

## Materials and methods

### Sampling

All the investigated samples or raw materials for making samples including pure samples or mixture of muscle tissues were prepared using commercially available lamb chops, pork legs, chicken drumsticks, laboratory rabbits, and mice. Sample H1 was made by mixing DNA from 6 different animal (Mutton, Beef, Rabbit, Rat, Pork and Chicken) with different mass percentages (Table 2). The samples SP-1, SP-2, and SP-3 were made by mixing sheep meat and pork meat with ratios of 99:1 (m/m), 90:10 (m/m) and 50:50 (m/m), respectively. The meat mass values of them are described in Table 3. For preparing the commercial samples, we bought frozen raw meat rolls and ham sausages produced by meat processing enterprises directly from the supermarkets.

### Laboratory reagents

CTAB (hexadecyl trimethyl ammonium bromide) lysis buffer was comprised of 2% (w/v) CTAB, 0.02 M EDTA, 0.1 M Tris (trihydroxyl methyl amino methane), and 1.4 M sodium chloride. This buffer was adjusted to pH 8.0 using 4 M hydrochloric acid and then autoclaved. The buffer used for DNA suspension was 1 M TE (Tris-EDTA) buffer (pH 8.0) from Sangon Biotech Co., Ltd. (Shanghai, China). The enzyme preparations of proteinase K, Premix Taq™ (TaKaRa Taq™ Version 2.0), Premix Ex Taq™ (Probe qPCR), and ROX plus were obtained from TaKaRa (Dalian, China). Chemicals and reagents, including CTAB, Tris, trichloromethane, isopentanol, absolute ethanol, were of at least analytical grade and were obtained from Sinopharm Group Co., Ltd. (Shanghai, China). The water used for this study was ddH_2_O (18 MΩ, UV sterilization).

### DNA extraction

Ground meat (2 gram) was added into a 15 mL tube. After adding 10 mL CTAB lysis buffer and 10 μL proteinase K solution (20 mg/L), the tube was incubated at 55 °C in a shaking incubator for 2 hours. A 2 mL tube containing 1 mL of lysate was centrifuged for 10 min at 12,000 × *g*. After transferring the supernatant to a new 2 mL tube, the same amount of trichloromethane: isopentanol: tris-phenol (24:1:25, v/v/v) was added. After overturn and blending, the tube was centrifugated for 10 min at 12,000 × *g*. The supernatant was transferred to a new 2 mL tube, and then the same amount of trichloromethane: isopentanol (24:1, v/v) was added. After overturn and blending, the tube was centrifugated for 10 min at 12,000 × *g*. The supernatant was moved to another new 1.5 mL tube, and then a two times volume of ice-cold absolute ethanol was added. After 30 min, the tube was centrifugated for 10 min at 12,000 × *g*. The precipitate was washed with 70% ethanol twice and then air-dried. Following this, 100 μL of TE buffer was added to the tube. After dissolution, the concentration and the quality of the extracted DNA were both determined by measuring absorbance at 260 nm (A_260_) and 280 nm (A_280_) using a micro spectrophotometer. The DNA concentration was calculated according to the following equation: c[ng/μL] = A_260_ × 50 × dilution factor. The ratio A_260_/A_280_ was used to assess the purity of the extracted DNA. The DNA extracts were stored at −20 °C before sequencing.

### High throughput sequencing

All the DNA sequencing experiments were performed by taking a commercial service provided by Majorbio Bio-Pharm Technology Co., Ltd (Shanghai, China). For HiSeq platform, DNA fragments with a length of around 150 bp were gotten by the ultrasonical disruption and followed purification of gel extraction. Paired-end library was constructed by adding the adaptors onto the fragments. Then, the sequencing was done following the introduction of Illumina. The softwares Seqprep and Sickle were used for quality-filtering of the obtained reads. As for MiSeq, DNA fragments of 385 bp were obtained using PCR amplification with universal primers. Then, purified amplicons were pooled in equimolar and paired-end sequenced (2 × 300) on an Illumina MiSeq platform (Illumina, San Diego, USA) according to the standard protocols by Majorbio Bio-Pharm Technology Co. Ltd. (Shanghai, China). Raw fastq files were quality-filtered by Trimmomatic and merged by FLASH with the standard criteria of the company. The tool FastQC (http://www.bioinformatics.babraham.ac.uk/projects/fastqc/) was used to evaluate the overall quality of both kinds of high throughput sequencing data. Only the data with good quality were used for the bioinformatics analysis. The average depth of coverage was calculated as previously described^32^.

### Construction of custom-built mitochondrial genome database

All the mitochondrial genome sequences which are available from NCBI were downloaded and saved as an XML format file. Then, a Perl script was run on Ubuntu 16.04 LTS for picking out all the sequences of the complete mitochondrial genomes, which belong to food producing animals, game animals, and meat adulteration related animals. The programed Perl script was shown in Supplementary information file (Script S1). The traditional classification of food producing animals was adopted for mitochondrial genomic sequence filtering, which is shown in Table S1. The resulting FASTA file which containing all the filtered mitochondrial genomic sequences was then indexed using the command “bowtie2-build” before being used for identification analysis.

### Phylogenetic analysis of *CYTB* amplicon

Amplicon sequences were spliced from the complete mitochondrial genomic sequences in GenBank with universal primers as the terminals. The software MEGA-X was used for multi-sequence alignment. The unweighted pair-group method with arithmetic means (UPGMA) was used for cluster analysis. The tree was constructed using MEGA-X by neighbor-joining method with Maximum Composite Likelihood and decorated with EvolView.

### PCR and real-time PCR

Three primer pairs for amplification of the *CYTB* gene, the *COI* gene, and the D-loop were obtained from the online system of the Barcode of Life Data System V4 (Bold Systems v4, http://www.boldsystems.org/), and the sequences are listed in Table 1. All primer and probe (Table 1) syntheses were performed by Thermo Fisher Scientific Inc. The reaction system and conditions of PCR and real-time PCR included a PCR reaction composed of 7.5 µL Premix Taq, 0.8 µL primers (100 mmol/L) each, 1 µL template DNA, and ddH_2_O added to 15 µL. PCR reaction steps included pre-degeneration at 94 °C for 5 min, followed by degeneration at 94 °C for 40 seconds, annealing at 60 °C for 40 seconds, and extension at 72 °C for 30 seconds for a total of 35 cycles. PCR products were separated by 2% agarose gel electrophoresis under 120 V constant pressure for 30 min.

### Identification analysis pipeline

The analysis pipeline described in Fig. 3 was achieved with Perl interpreter on the Ubuntu 16.04 LTS. The programed script was provided in the Supplementary information file (Script S2), which can be copied into a TXT file and run directly. The software Bowtie 2.2.2.9 was introduced for mapping the obtained short reads to mitochondrial genomes deposited in the custom-built database. The parameters for Bowtie alignments were set as default in the Perl script. The file format of output file was set as SAM. After getting the SAM file, it was then analyzed with a line-by-line manner to count up the numbers of unique reads. The unique read was defined as the read that can only map to genomes belong to a single meat category.

### Sequencing throughput optimization

To determine the lower limit of the sequencing data size, simulated sequencing fastq files containing less reads were generated by randomly extracting reads from a raw Illumina sequencing fastq file. The extracting rates were set as 100%, 62.5%, 12.5%, 2.5%, 0.5%, and 0.1%, respectively. For each extracting rate, the read extraction was repeated 4 times. A Per script was programed for doing the read extraction (Script S3). The obtained simulated sequencing result files were then used as standard inputs for identification analysis to investigate the method accuracy and standard division.

## Supplementary information

Supplementary information.

## References

[1] Ballin NZ (2010). Authentication of meat and meat products. Meat Sci..

[2] Nakyinsige K, Man YB, Sazili AQ (2012). Halal authenticity issues in meat and meat products. Meat Sci..

[3] Staats M (2016). Advances in DNA metabarcoding for food and wildlife forensic species identification. Anal. Bioanal. Chem..

[4] Shokralla S, Spall JL, Gibson JF, Hajibabaei M (2012). Next-generation sequencing technologies for environmental DNA research. Mol. Ecol..

[5] Coghlan ML (2012). Deep sequencing of plant and animal DNA contained within traditional Chinese medicines reveals legality issues and health safety concerns. PLoS Genet..

[6] Pompanon F (2012). Who is eating what: diet assessment using next generation sequencing. Mol. Ecol..

[7] Kumar G, Rehman F, Chaturvedy V (2017). Soft Tissue Applications of Er,Cr:YSGG Laser in Pediatric Dentistry. Int J Clin Pediatr Dent.

[8] Teletchea F, Maudet C, Hanni C (2005). Food and forensic molecular identification: update and challenges. Trends Biotechnol..

[9] Matsunaga T (1998). Determination of mitochondrial cytochrome B gene sequence for red deer (Cervus elaphus) and the differentiation of closely related deer meats. Meat Sci..

[10] Matsunaga T (1999). A quick and simple method for the identification of meat species and meat products by PCR assay. Meat Sci..

[11] Wolf C, Rentsch J, Hübner P (1999). PCR-RFLP analysis of mitochondrial DNA: a reliable method for species identification. J. Agric. Food Chem..

[12] Partis L (2000). Evaluation of a DNA fingerprinting method for determining the species origin of meats. Meat Sci..

[13] Tobe SS, Linacre AM (2008). A multiplex assay to identify 18 European mammal species from mixtures using the mitochondrial cytochrome b gene. Electrophoresis.

[14] Jia X (2015). Investigation of genetic diversity and evolution of Tibetan chicken based on complete mitochondrial DNA D-loop region sequence. J. Northeast Agric. Univ..

[15] Karabasanavar N, Girish PS, Kumar D, Singh SP (2017). Detection of beef adulteration by mitochondrial D-loop based species-specific polymerase chain reaction. Int. J. Food Prop..

[16] Qiao Ge-Xia, WANG Jianfeng, Jiang Li-Yun (2011). Use of a mitochondrial COI sequence to identify species of the subtribe Aphidina (Hemiptera, Aphididae). ZooKeys.

[17] Kocher TD (1989). Dynamics of mitochondrial DNA evolution in animals: amplification and sequencing with conserved primers. Proc. Natl. Acad. Sci. U. S. A..

[18] Bravi CM (2004). A simple method for domestic animal identification in Argentina using PCR-RFLP analysis of cytochrome b gene. Leg. Med. (Tokyo).

[19] Murugaiah C (2009). Meat species identification and Halal authentication analysis using mitochondrial DNA. Meat Sci..

[20] Verma SK, Singh L (2003). Novel universal primers establish identity of an enormous number of animal species for forensic application. Mol. Ecol. Notes.

[21] Tillmar AO, Dell’Amico B, Welander J, Holmlund G (2013). A universal method for species identification of mammals utilizing next generation sequencing for the analysis of DNA mixtures. PLoS One.

[22] Ripp F (2014). All-Food-Seq (AFS): a quantifiable screen for species in biological samples by deep DNA sequencing. BMC Genomics.

[23] Simon C, Frati BF, Stewart JB, Beckenbach AT (2006). Incorporating molecular evolution into phylogenetic analysis, and a new compilation of conserved polymerase chain reaction primers for animal mitochondrial DNA. Annu. Rev. Ecol. Evol. Syst..

[24] Langmead B (2010). Aligning short sequencing reads with Bowtie. Curr. Protoc. Bioinform..

[25] Galimberti A (2013). DNA barcoding as a new tool for food traceability. Food Res. Int..

[26] Lü Dongmei HY, Hui WEN, Xiaoling ZHAO (2015). HUANG Chong. Application of DNA barcoding in authentication of food product. Food Sci..

[27] Bang-Hui MO (2008). DNA barcoding identification I. Research progress and applied perspective of DNA barcoding. Sichuan J. Zoo.

[28] Ling S, Yajun WU, Han J, Qiao X, Chen Y (2017). A review of recent advances in the application of DNA barcoding in identification of animal species in highly processed animal-derived products. Meat Res.

[29] Kwong S, Srivathsan A, Meier R (2012). An update on DNA barcoding: low species coverage and numerous unidentified sequences. Cladistics.

[30] Kitano T, Umetsu K, Tian W, Osawa M (2007). Two universal primer sets for species identification among vertebrates. Int. J. Legal Med.

[31] Morey M (2013). A glimpse into past, present, and future DNA sequencing. Mol. Genet. Metab..

[32] Churchill JD (2015). Blind study evaluation illustrates utility of the Ion PGM™ system for use in human identity DNA typing. Croat. Med. J..

