# Supplementary material for: Simultaneous identification of animal-derived components in meats using high-throughput sequencing in combination with a custom-built mitochondrial genome database
Source: Sci Rep. 2020 Jun 2;10:8965. doi: 10.1038/s41598-020-65724-4 (PMC7265478; doi:10.1038/s41598-020-65724-4)
Supplement: Supplementary file 1 — Supplementary information. [file 41598_2020_65724_MOESM1_ESM.docx]

**Supplementary Information**

Biotechnology

Simultaneous identification of animal-derived components in meats using high-throughput sequencing in combination with a custom-built mitochondrial genome database

Yi-nan Zhang^1§^, Qinfeng Qu^1^, Mingzhen Rao^3^, Na-na Zhang^3^, and Yu Zhao^3^, Fei Tao^2§^

1 Shanghai Institute of Quality Inspection and Technical Research, Shanghai 200233, People’s Republic of China.

2 State Key Laboratory of Microbial Metabolism, and School of Life Sciences & Biotechnology, Shanghai Jiao Tong University, Shanghai 200240, People’s Republic of China.

3 College of Life Science, Shanghai Normal University, Shanghai 200234, People’s Republic of China.

^§^Correspondence: Yi-nan Zhang and Fei Tao.

Mailing address: Shanghai Institute of Quality Inspection and Technical Research, Cangwu Road No. 381, Shanghai 200233, People’s Republic of China.

E-mail: zhangyn@sqi.org.cn, taofei@sjtu.edu.cn.

Tel: +86-021-54265930; Fax: +86-021-64852831.

**Supplementary Tables**

**Table S1．Corresponding relation between meat categories and taxonomic genus**

| **category of Meat** | **Genus** |
| --- | --- |
| Pork | *Sus* |
| Beef | *Bos, Bison, Syncerus, Bubalus* |
| Mutton/lamb | *Capra, Ovis, Pseudois* |
| Rabbit meat | *Lepus, Oryctolagus, Pentalagus, Sylvilagus, Romerolagus* |
| Venison | *Cervus, Capreolus, Odocoileus, Muntiacus, Mazama, Rusa, Hydropotes* |
| Chicken | *Gallus* |
| Horse meat | *Equus* |
| Rat meat | *Mus, Apodemus, Rattus, Micromys, Meriones, Acomys, Melomys, Tokudaia, Niviventer, Gerbillurus, Pseudomys, Leggadina, Leopoldamys, Uromys, Rhombomys, Brachiones, Diplothrix* |
| Dog meat | *Canis* |
| Camel meat | *Camelus, Vicugna* |
| Fox meat | *Urocyon, Vulpes* |
| Cat meat | *Felis, Panthera, Prionailurus, Lynx* |
| Bear meat | *Ursus, Helarctos, Ailuropoda, Melursus, Tremarctos, Arctodus* |

**Table S2．Sample information and detection results of commercial meat products**

| Sample ID | Sample information | | Detection results of | | |
| --- | --- | --- | --- | --- | --- |
|  | name | main ingredient^&^ | | NGS  (percentage of mapped reads ) | Fluorescence PCR  (Ct value) |
| SQI-P1-1 | Beef slices | beef | | Beef (99.19%) | Bovine+(21.19) |
| SQI-P1-2 | Lamb slices | lamb | | Mutton (96.79%) | Ovis +(17.16) |
| SQI-P1-3 | Mutton rolls | mutton and salt | | Mutton (98.51%) | Ovis+(16.48) |
| SQI-P1-4 | Lamb rolls | lamb and salt | | Mutton (95.76%)  Pork (3.10%) | Ovis+(16.55)  Porcine-(38.99) |
| SQI-P1-5 | Mutton rolls | mutton and salt | | Mutton (97.40%)  Chicken (1.09%) | Ovis+(16.87)  Chicken+(20.08) |
| SQI-P1-6 | Mutton slices | not mentioned (bulk) | | Mutton (97.93%) | Ovis+(16.57) |
| SQI-P2-1 | Ham sausage for noodle soup | pork, chicken, starch, and etc. | | Chicken (72.65%)  Pork (26.97%) | Chicken+(18.06)  Porcine+(31.06) |
| SQI-P2-2 | Chicken ham sausage | chicken, pork, starch, soybean protein, eggs, and etc. | | Chicken (78.05%)  Pork (21.76%) | Chicken+(17.20)  Porcine+(31.35) |
| SQI-P2-3 | Ham sausage | chicken, pork, and etc. | | Pork (69.69%)  Chicken (30.08%) | Chicken+(17.20)  Porcine+(31.07) |
| SQI-P2-4 | Spicy and crispy ham sausage | chicken, pork, and etc. | | Chicken (55.05%)  Pork (44.69%) | Chicken+(16.25)  Porcine+(29.55) |
| SQI-P2-5 | Beef-flavored Muslim Sausage | chicken, beef, starch and etc. | | Chicken (99.14%)  Beef (0.56%) | Chicken+(17.16)  Bovine-(>40) |
| SQI-P2-6 | Flavored chicken sausage | chicken, starch, and etc. | | Chicken (99.11%) | Chicken+(16.79) |
| SQI-De-1* | Beef rolls | beef | | Beef (97.26%) | Bovine+(23.18) |
| SQI-De-2* | Beef rolls | beef | | Beef (96.69%) | Bovine+(22.28) |
| SQI-De-3* | Beef rolls | beef | | Beef (98.77%) | Bovine+(22.86) |
| SQI-De-4* | Beef rolls | beef | | Beef (95.14%) | Bovine+(17.70) |
| SQI-De-5* | Beef rolls | beef | | Beef (97.34%) | Bovine+(18.72) |
| SQI-De-6* | Beef rolls | beef | | Beef (93.30%) | Bovine+(17.92) |
| SQI-3-1 | Beef stick | beef, maltose, sugar, and etc. | | Beef (99.97%) | Bovine+(16.18) |
| SQI-3-2 | Satay flavor beef jerky | beef, sugar, salt, and etc. | | Beef (99.84%) | Bovine+(16.00) |
| SQI-3-3 | Spicy beef jerky | beef, sugar, salt, etc. | | Beef (70.79%)  Pork (25.86%) | Bovine+(14.22)  Porcine-(37.97) |
| SQI-3-4 | Pork floss | pork hind leg meat, soy sauce, sugar, and etc. | | Pork (81.93%)  Chicken (18.07%) | Porcine+(23.59)  Chicken+(17.09) |
| SQI-4-1 | Stewed chicken | chicken, salt, brewing soy sauce, yellow wine, scallion, gourmet powder, spice | | Chicken (95.00%)  Pork (4.99%) | Chicken+(16.50)  Porcine- (38.06) |
| SQI-4-2 | Baked Pork Chops | pork, soy protein, starch, salt, sugar, egg white powder, soy sauce, spice and etc. | | Pork (100.00%) | Porcine+(21.93)  Chicken+(27.71) |
| SQI-4-3 | Lunch leg sausage | chicken, food additives, pork, maltose, soy protein, salt, sugar | | Pork (57.40%)  Chicken (42.60%) | Porcine+(24.94)  Chicken+(16.11) |
| SQI-4-4 | Sliced ham | Pork, food additives, glucose, soy protein, salt | | Pork (100.00%) | Porcine+(23.17) |
| SQI-4-5 | soy sauce spiced pork | pork, hog skin, chicken, soybean protein with spicy soybean sauce, maltose, sugar, salt | | Pork (96.74%)  Chicken (3.25%) | Porcine+(23.84)  Chicken+(18.60) |
| SQI-4-6 | American ham | pork, glucose, salt | | Pork (100.00%) | Porcine+(23.87) |

Note: & From ingredient table; * DNA extracted everyday from the minced comercial beef rolls which were allowed to stand at room temperature for 6 days; + for positive results, - for negtive results

Table S3. Identification using non-amplification method

|  | **True DNA amount of Sample I** | | **Deep sequenceing analysis** | | **Deviation** | |
| --- | --- | --- | --- | --- | --- | --- |
|  | **DNA (ng/µL)** | **True proportion** | **matching number** | **measured**  **proportion** | **abs.** | **rel.** |
| **Pork** | 32.87 | 9.03% | 1180 | 8.75% | 0.28% | 3.10% |
| **Beef** | 111.95 | 30.76% | 5292 | 39.23% | 8.47% | 27.54% |
| **Mutton** | 213.63 | 58.71% | 6941 | 51.45% | 7.26% | 12.37% |
| **Chicken** | 5.45 | 1.50% | 66 | 0.49% | 1.01% | 67.33% |
| **Rabbit** | 0 | 0.00% | 4 | 0.03% | - | - |
| **Venison** | 0 | 0.00% | 7 | 0.05% | - | - |

The simulated sample was prepared by mixing 4 kinds of DNA which was extracted from different meats. Then the DNA was used for library construction and sequencing on HiSeq platform. We obtained totally 9,212,504 reads with no poor-quality ones. The read length is 151 bp. The percent of GC is 42%. After identification analysis, the difference of the measured and true value was compared in the table. There are four kinds of DNA in the simulated sample including the DNA from pork, beef, mutton and chicken. All the four meat components can be detected while there are 2 false positives. The matching numbers of the two false-positive categories, rabbit and venison, is 4 and 7, which is 0.03% and 0.05% of the whole matching number, respectively. The difference of the proportion between the true value and the measured one in other four kinds of DNA was compared by absolute and relative deviation calculation. The absolute deviation is arranged from 0.28% to 8.47%, and the relative deviation from 3.10% to 67.33%. The deviation is may be caused by the different contents of mitochondria in different meats.

**Supplementary Figures**

**
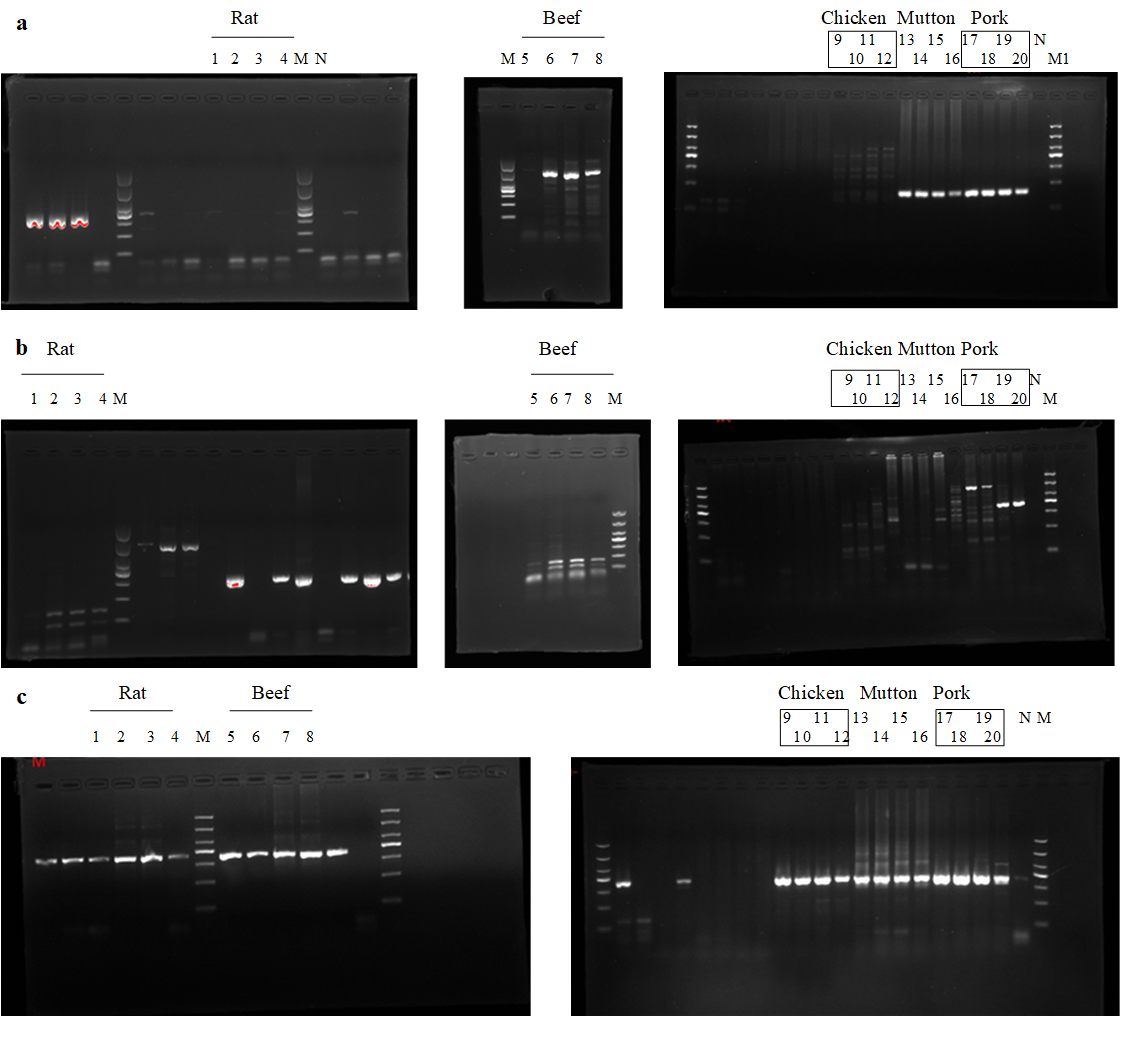
**

**Figure S1: Agarose gel electrophoresis of products amplified using universal primers**

Electrophorogram a is the COI-P5 amplicon; electrophorograms b is the D-loop amplicon; electrophorograms c is the CYTB amplicon; Templates corresponding lanes include: 1-4, rat; 5-8, beef; 9-12, chicken; 13-17, Mutton ; 18-21, pork; 22, Negative control; M, Marker DL 1000; M1, Marker DL15000. The annealing temperatures of lanes in each template group are 50°C, 55°C, 60°C, and 65°C from left to right.


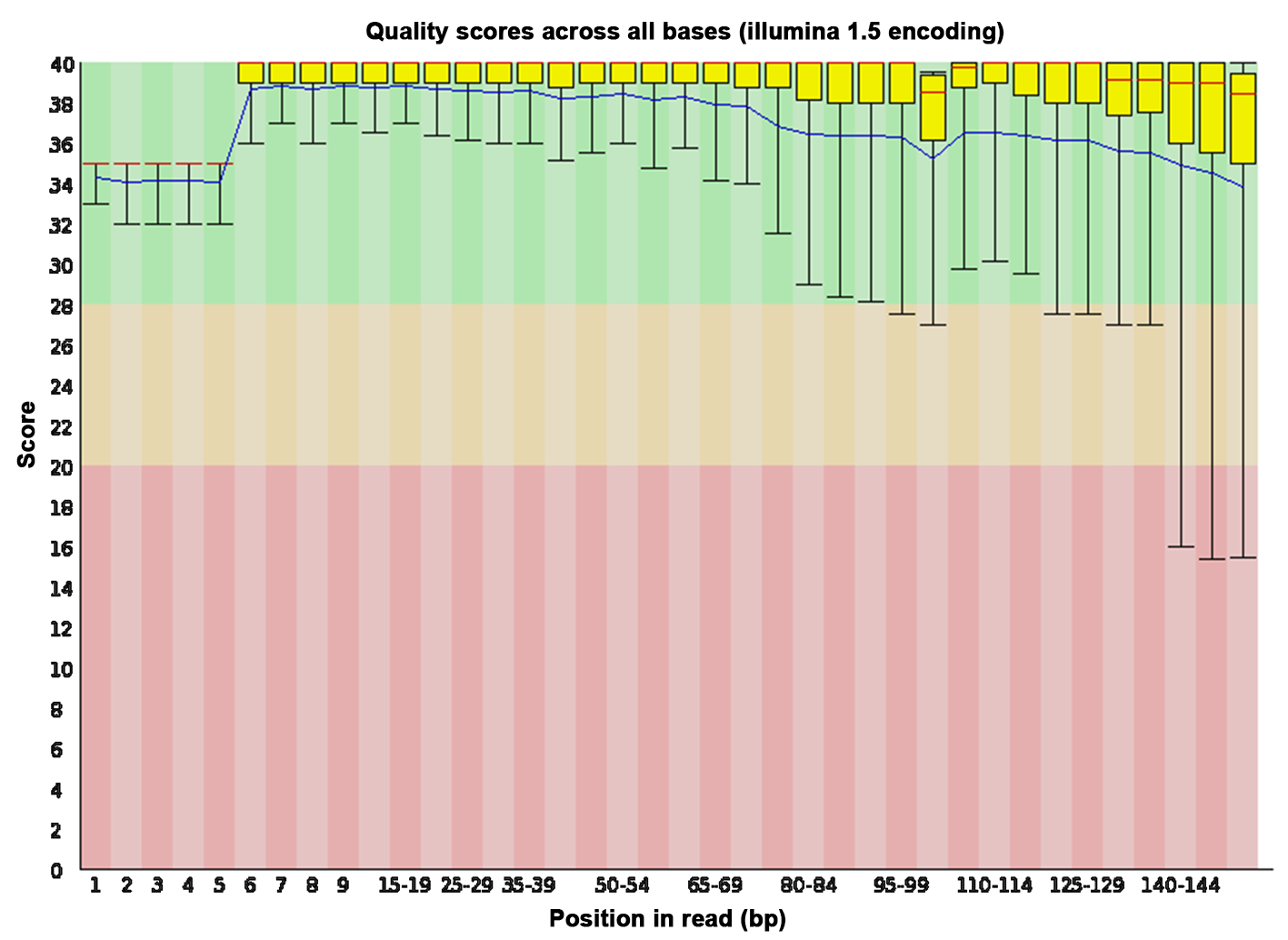


**Figure S2: Per base sequence quality of CYTB gene sequencing**

The Fred value was used to present the quality (X axis). It was calculated using the formula -10*log10(p), where the p represents the probability of sequencing errors. The Y axis represents the position in a sequencing read. The red is the median, the yellow is the 25% to 75% range, the tentacles are the 10% to 90% range, and the blue line is the average. A “WARN” should be reported if the lower quartile of any position is less than 10 or if the median is less than 25. If the lower quartile in any position is less than 5 or if the median is less than 20, a "FAIL" should be reported.


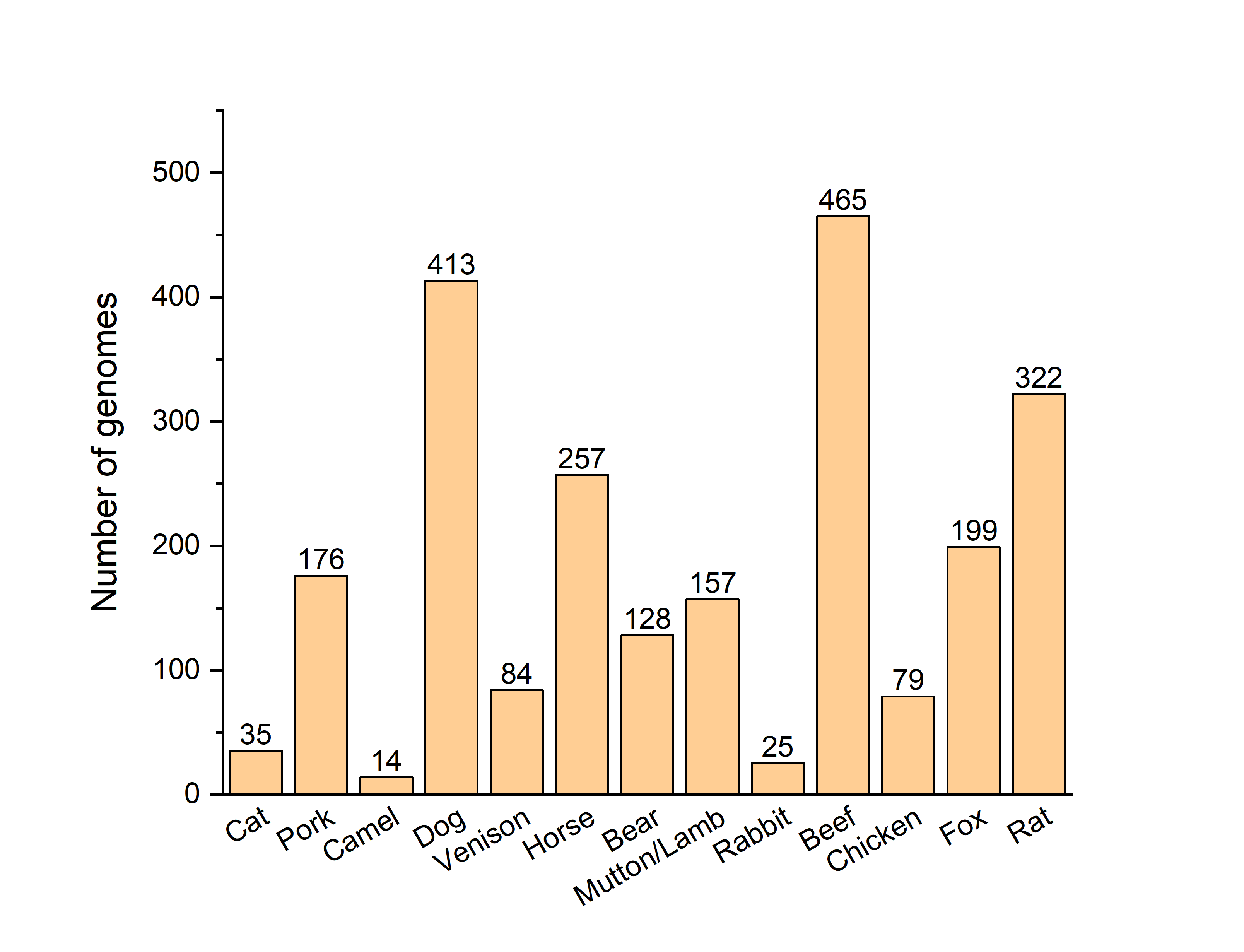


**Figure S3: Mitochondrial genomes in the custom-built database**

After filtering, the numbers of complete mitochondrial genomes belong to different meat types were counted. The numbers are also shown the figure directly.

**Supplementary scripts:**

**Script S1: filter.pl**

#! /usr/bin/perl -w

###############################################################################

# This script is for picking out the mitochondrial genomic sequences from an XML file

# downloaded from NCBI.

# The filtering mainly based on the sequencing status of genome and provided genus names.

# For running the script an XML file containing genomic sequences and an xlsx format file

# need to be provided as the inputs.

# The output of this script is a FASTA file which contain all the target mitochondrial genomic

# sequences.

# The IDs of each sequence in the output FASTA file is as follows:

# “>meat classification name|genus name|`accession number in NCBI”

# Programed by Fei Tao (taofei@sjtu.edu.cn). Copyright reserved.

###############################################################################

############################## Modules ######################################

use strict;

use Bio::Seq;

use Bio::SeqIO;

use Cwd qw(abs_path);

use FindBin qw($Bin);

use lib "$Bin/../lib";

use XML::Simple;

use Data::Dumper;

use Spreadsheet::ParseXLSX;

############################## Main #######################################

## Read input

my $infile = shift;

my $infile2 = shift;

open In, "$infile" or die "Cann't open the input file";

my $outfile = $infile;

$outfile =~ s/.+\///;

$outfile =~ s/\..+//;

my $outseq = Bio::SeqIO->new(-file => ">$outfile.fa",

-format => 'fasta');

## Read the sheet of animals' names

my $parser = Spreadsheet::ParseXLSX->new;

my $book = $parser->parse($infile2);

if ( !defined $book ) {die $parser->error(), ".\n";}

my $sheet = $book->{Worksheet}->[0];

## Create a hash for the genus names

my $hash = {};

for (my $r=0;$sheet->get_cell($r,1);$r++){

my $name_eng = $sheet->get_cell($r,1)->value;

$name_eng =~ s/\s//g;

my @names_spec;

for (my $c=2;$sheet->get_cell($r,$c)&&$sheet->get_cell($r,$c)->value;$c++){

my $name_spec = $sheet->get_cell($r,$c)->value;

$name_spec =~ s/\s//g;

push @names_spec,$name_spec;

}

@{$hash->{$name_eng}} = @names_spec;

#print @{$hash->{$name_eng}},"\n";

}

## Treat the database, screen the sequences one by one

my $number = -1; # Set as a positive integer for testing, and as -1 for filtering

my $string;

my $count;

while(<In>){

$string .= $_;

my @entries = $string =~ /\<TSeq\>.*?\<\/TSeq\>/mgs;

$string =~ s/.*?\<TSeq\>.*?\<\/TSeq\>//mgs;

foreach my $entry (@entries){

#####################################################

# Analyze the xml entry one by one

my $xml = new XML::Simple;

my $data = $xml->XMLin($entry, ForceArray => 1, KeyAttr => []);

print $data->{'TSeq_gi'}->[0],"\n" if $data->{'TSeq_gi'}->[0]; # Print on screen

if ($data->{'TSeq_orgname'}->[0] && $data->{'TSeq_defline'}->[0]){

if ($data->{'TSeq_defline'}->[0] =~ /complete genome/gi){

for my $animal (keys %{$hash}){

for my $speci(@{$hash->{$animal}}){

if ($data->{'TSeq_orgname'}->[0] =~ /^$speci/gi){

my $id = $animal."|".$data->{'TSeq_orgname'}->[0]."|".$data->{'TSeq_gi'}->[0];

$id =~ s/\s+/_/gi;

my $seq = $data->{'TSeq_sequence'}->[0];

my $stream = Bio::Seq->new( -seq => $seq,

-id => $id,

);

$outseq->write_seq($stream);

}

}

}

}

}

######################################################

$number--;

last if $number == 0;

}

last if $number == 0;

}

close In;

**Script S2: analysis.pl**

#!usr/bin/perl -w

use strict;

###############################################################################

# This script is programed for analysis fastq files obtained from high throughput sequencing.

# One need to provide a name of the folder which contains at least 2 fastq files (pair-end)

# The output of this script is a TSV format file which contains the calculated percentages of

# different meat components.

# Programed by Fei Tao (taofei@sjtu.edu.cn). Copyright reserved.

###############################################################################

######################## Main ##############################################

## Get all the file names in the target folder

my $indir = shift;

$indir =~ s/\\$//; #delete the "\" in the end of dir name

$indir =~ s/\/$//; #delete the "/" in the end of dir name

opendir(DIR,"$indir"|| die "can't open this $indir");

my @files =readdir(DIR);

closedir(DIR);

my %samples;

foreach my $file (@files){

$file =~ /(.*)_\d\.fq$/;

$samples{$1} = ();

}

my @files_2 = sort keys(%samples);

## Treat the files one by one

foreach my $sample (@files_2){

######### Run Bowtie2 ###############

my $input1 = $indir.'/'.$sample.'_1.fq';

my $input2 = $indir.'/'.$sample.'_2.fq';

my $rf = '~/tf/mitochondrion/rf/mito';

system "bowtie2 -p 4 -x $rf -1 $input1 -2 $input2 -S $sample.sam ";

#Count the numbers and output final results

my $infile = "$sample.sam";

my $outfile = "$sample.csv";

print $infile, "\n"; # Screen output

open In, $infile or die "can not open";

open Out, ">$outfile" or die;

my %hash;

my $c = -1;

while(<In>){

chomp;

unless(/^@/){

my ($qname, $flag, $rname, $pos,

$mapq, $cigar, $rnext, $pnext, $tlen, $seq, $qual) = split(/\t/);

unless ($rname eq '*'){

my @tem = split(/\|/,$rname);

my $hit = $tem[0];

$hash{$qname} .= "$hit," unless ($hash{$qname} &&

$hash{$qname} =~ /$hit/gi);

}

}

last if $c-- == 0;

}

close In;

#delete hashes which can mate to more than one animal

for my $reads (keys %hash){

chop $hash{$reads};

my @hits = split(/,/, $hash{$reads});

delete $hash{$reads} if @hits > 1;

}

#count the hits of each animal

my %output;

for my $reads (keys %hash){

$output{$hash{$reads}}++;

}

#output the results

for my $a (keys %output){

print Out $a,",", $output{$a},"\n";

}

close Out;

}

**Script S3: extract.pl**

#! /usr/bin/perl -w

use strict;

###############################################################################

# This script is programed for extracting reads from a fastq file.

# A file of fastq is needed as the input

# The output of this script is a series of fastq files

# Programed by Fei Tao (taofei@sjtu.edu.cn). Copyright reserved.

###############################################################################

die "#usage:perl $0 <fq><threshold>/n" unless @ARGV==1;

my $fa=shift;

my @thr = (0.001, 0.005, 0.025, 0.125, 0.625); #Set the extraction rate here

foreach my $threshold (@thr){

my $name1 = "extract$fa".($threshold*100)."_1";

my $name2 = "extract$fa".($threshold*100)."_2";

open Out1, ">$name1.fq";

open Out2, ">$name2.fq";

my $num = $fa=~/\.gz/? `gzip -cd $fa|wc -l`/8 :`less $fa|wc -l`/8;

my $need=int($num*$threshold);

my %ha;

for(;;){

my $tmp=int(rand($num));

$ha{$tmp}=1;

last if (keys %ha)==$need;

}

$fa=~/\.gz/?(open IN,"gzip -cd $fa|"||die) : (open IN,$fa||die);

while(<IN>){

chomp;

my $line=($.-1)/8;

if(exists $ha{$line}){

print Out1 "$_\n";

print Out1 scalar <IN> for 1..3;

print Out2 scalar <IN> for 4..7;

}

}

close IN;

close Out1;

close Out2;

}
